# Study of the Properties and Modification Mechanism of SBS-Modified Asphalt by Dry Process

**DOI:** 10.3390/ma17071454

**Published:** 2024-03-22

**Authors:** Ying Wang, Shaohua Guo, Zhongshi Pei, Shizuo Zhan, Senlin Lin, Kezheng Ma, Junwen Lei, Junyan Yi

**Affiliations:** 1 School of Transportation Science and Engineering, Harbin Institute of Technology, No. 73 Huanghe Road, Nangang District, Harbin 150090, China; hitwang@stu.hit.edu.cn (Y.W.); 13946761923@163.com (K.M.); junwen.lei@foxmail.com (J.L.); 2 Liaoning Provincial Transportation Planning and Design Institute Co., Ltd., No. 42, Lidao Road, Heping District, Shenyang 110166, China; guoshaohuaji@126.com (S.G.); zuo_fantasy@163.com (S.Z.); lsl421357732@163.com (S.L.)

**Keywords:** pavement, dry process, modified asphalt binder, performance, mechanism

## Abstract

SBS (styrene-butadiene-styrene block copolymer) is a thermoplastic elastomer with properties most similar to rubber. SBS asphalt modifier is mainly composed of a styrene-butadiene-styrene block copolymer with a certain amount of additives and stabilizers. SBS-modified asphalt binder has always been the most commonly used pavement material both domestically and internationally. However, conventional wet-process SBS-modified asphalt binder requires manufacturers to produce it in advance and transport it to a mixing plant for blending. This has provided an opportunity for unscrupulous businesses to reduce the amount of SBS by adding other substances, allowing inferior asphalt binder to pass inspections undetected. At the same time, conventional wet-process SBS-modified asphalt tends to undergo phase separation and experience a decline in performance as the storage time increases. However, dry-process SBS-modified asphalt can be directly added at the mixing plant, effectively addressing the issues associated with conventional wet-process SBS-modified asphalt. It also helps to reduce environmental pollution to a certain extent. This study investigates the extraction process of dry-process SBS-modified asphalt binder. It clarifies the performance and modification mechanisms of two types of dry-process SBS-modified asphalt binder at different dosages through various testing methods, including basic indicators, rheological properties, infrared spectroscopy, and fluorescence microscopy. The results indicate that due to the incorporation of oil, crosslinker, solubilizer, and other substances into dry-process SBS modifier, there is a small amount of chemical reaction with asphalt in the melting process. The high- and low-temperature properties and fatigue properties of the two dry-process SBS-modified asphalt binders at a 7% dosage are close to wet SBS-modified asphalt binder at a 5% dosage.

## 1. Introduction

The wet modification process is to use virgin asphalt binder as a raw material in a mixing station, add a certain proportion of modifier, use large asphalt shear or colloid mill equipment, make the modifier swell and evenly dispersed in the asphalt binder to form a blend material, and then mix with aggregate to produce a modified asphalt mixture [1]. SBS-modified asphalt binder is generally processed and manufactured by a factory using the wet process; the construction technology is mature, and it is the most widely used pavement material [2,3,4]. However, in engineering practice, there are often many problems with SBS-modified asphalt binder obtained by the wet process. For example, finished SBS-modified asphalt binder supplied by plants has storage stability problems due to modifier segregation [5]. Due to the degradation of modified asphalt with the development of high-temperature shear during processing, the performance of modified asphalt will decay from preparation to actual use, which will affect the performance of the mixture [6,7]. On the other hand, the amount of SBS in wet SBS-modified asphalt cannot be accurately tested at the construction site. SBS content in SBS-modified asphalt generally needs to be detected by infrared spectroscopy, but this method is not suitable for on-site detection [8]. At present, the performance of finished asphalt is generally tested by measuring the penetration degree, softening point, ductility, and other indicators in project management, which allows some “unscrupulous businesses” to have the opportunity to cope with the test by adding certain additives and reducing the amounts of modifiers to seek more benefits for themselves. The difficulty of quantitative testing of these indicators increases the difficulty of supervision. The reasons for the above problems are not only the objective nature of SBS-modified asphalt but also the problem that it is difficult for manufacturers to supervise. In addition, wet modification also has problems such as large energy consumption and environmental pollution.

In order to solve the above problems from the root, dry modification is gradually being developed and applied. The biggest difference from the wet modification process is that the dry process is to directly produce a modified asphalt mixture by placing the modifier, asphalt binder, and aggregate into a mixing building, respectively, realizing “mixing is modification”. It can be ensured that storage materials do not deteriorate, and the quality and dosage of each material are transparently developed, which can enable manufacturers to reduce the cost of production equipment and transportation and solve the problems of unstable quality of conventional wet SBS-modified asphalt [9,10]. At the same time, dry modification can effectively improve pollution in the asphalt production process and respond to national energy conservation and environmental protection sustainable development strategies.

Cardone F et al. studied the effect of polyolefin additives (POs) on the mechanical response of modified asphalt concrete by dry and wet processes, and the results showed that the dry process had better deformation resistance and better aging resistance than the modified asphalt mixture produced by the wet process [11]. Dong et al. used molecular dynamics simulations to study the diffusion behavior of dry-modified SBS at the asphalt–aggregate interface. The results show that the diffusion distribution of dry-modified asphalt is similar to that of wet-process SBS-modified asphalt with sufficient temperature and time [12]. Rondon Quintana presented the laboratory results of tests performed on samples of Gilsonite-modified hot-mix asphalts modified by wet and dry processes. The result showed that a modified asphalt mixture under the dry process developed lower strength and stiffness under monotonic and dynamic loading [13]. A study by Xu et al. showed that the road performance of an SBSM (multi-component SBS)-modified asphalt mixture under dry modification is slightly inferior to wet modification, but satisfies engineering requirements [14]. A study by Liang et al. showed that a dry-processed rubber-modified asphalt pavement has better pavement performance compared to a conventional asphalt pavement [15]. Nielsen studied the process of SBS and clastic rubber dry-modified asphalt, carried out tests and reference sections on Danish motorways, and took samples from the field for high- and low-temperature properties, creep, fatigue, and other properties. The results showed that the application was good [16]. Germany has developed Duroflex dry-process modifier products that could replace SBS modifiers. Some scholars have conducted research on its performance, combined with engineering practice, and proved the advantages of this additive in a large number of test data and amount of use experience. Gao’s study found that the high-temperature performance, low-temperature performance, water stability, and dynamic modulus of a Duroflex asphalt mixture are significantly higher than for an SBS-modified asphalt mixture and an AH-90 asphalt mixture, which suggests that Duroflex significantly improves the road performance of asphalt mixtures [17]. Mahmoudi investigated the influence of crumb rubber added by the dry process on the dissipative properties of bituminous mixtures. Results show that pavements made with bituminous mixtures containing crumb rubber added by the dry process dissipate more energy with respect to reference mixtures without crumb rubber [18]. Baumgardner’s study showed that dry-process rubber-modified asphalt mixtures can lead to easier mixing, reduced tackiness, reduced cracking, less permanent deformation, lower life-cycle cost, and longer service life [19].

At present, there are a few studies on dry-modified asphalt binder and mixture, mainly focusing on the process, but the modification mechanism of dry modifiers still needs to be explored. Therefore, in this study, two dry SBS modifiers were used to prepare an asphalt mixture; the asphalt in the dry SBS-modified asphalt mixture was extracted by a specific process, and the properties and modification mechanism were studied. This study provides a new idea for the process optimization of dry SBS-modified asphalt mixture and lays a foundation for the large-scale application of dry-modified asphalt mixture.

## 2. Materials and Test Methods

### 2.1. Virgin Asphalt Binder

In this study, Liaohe Petrochemical 90# virgin asphalt binder from Northeast China was used. Referring to the “Standard Test Methods of Bitumen and Bituminous Mixtures for Highway Engineering” (JTG E20 2011) [20], the index test results of the Liaohe 90# virgin asphalt binder are shown in Table 1 (Two sets of parallel samples were tested).

### 2.2. SBS Modifier

In this study, the basic technical indexes of conventional wet modifiers are shown in Table 2, and the basic technical indexes of Liaohe brand and Guolu brand dry SBS modifiers are shown in Table 3.

### 2.3. Test Methods

#### 2.3.1. Fourier Infrared Spectrometer (FTIR)

**Figure 1:**
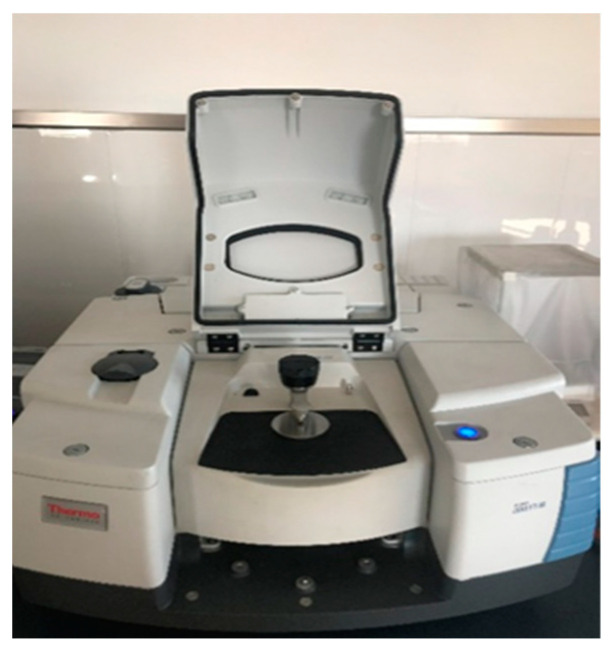
FTIR instrument.

The test instrument was a Thermo Fisher Nicolet iS10 (Waltham, MA, USA) infrared spectrometer. By using attenuated total reflection infrared spectroscopy (ATR), solid asphalt samples can be scanned directly, and the reflected signals on the surface can be collected, so as to analyze the organic components and the structure information of inorganic substances on the surface. The resolution was set to 4 cm^−1^, the scan was carried out 32 times, and the test range was 4000~500 cm^−1^. Two parallel samples were prepared for each sample, and each sample was scanned twice. The instrument is shown in Figure 1.

#### 2.3.2. Dynamic Shear Rheometer (DSR)

**Figure 2:**
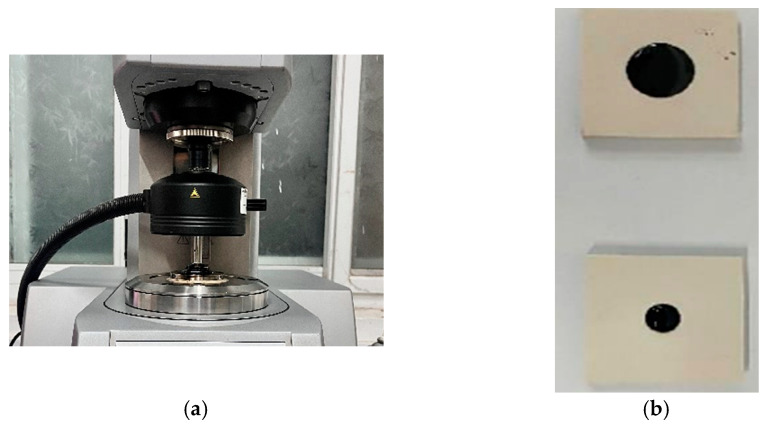
DSR test instrument and asphalt binder samples. (**a**) DSR test equipment. (**b**) Samples.

A DSR (Anton Paar, Smart pave 102, Graz, Austria) is a precision analytical instrument used to study the rheological properties of asphalt. The instrument can be used for SHRP (Strategic Highway Research Program) grading measurement, multi-stress creep measurement, strain scanning measurement, viscosity measurement, etc. It can be used to measure virgin asphalt, polymer-modified asphalt, and aged asphalt. DSR’s parallel test system consists of 25 mm or 8 mm diameter plates with 1.00 mm or 2.00 mm test clearances, respectively. In this study, two parallel samples were prepared for each sample. The test instrument and asphalt samples are shown in Figure 2.

#### 2.3.3. Bending Beam Rheometer (BBR)

**Figure 3:**
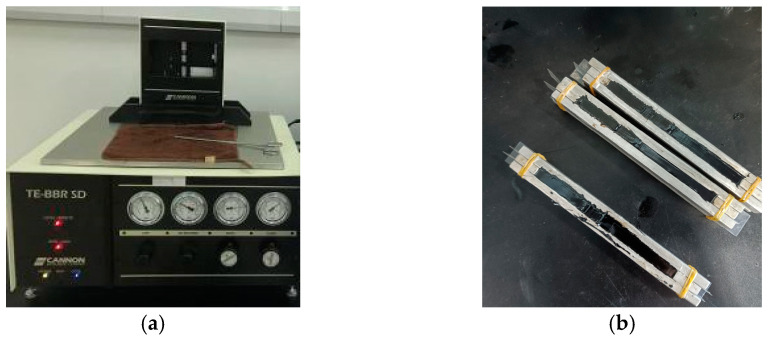
BBR test instrument and asphalt samples. (**a**) BBR test equipment. (**b**) Samples.

A BBR (SYD-0627, Shanghai Changji Geological Instrument Co., Ltd., Shanghai, China) is a test instrument used to measure the stiffness and stress relaxation rate of materials. During the BBR test, the trabecular specimen size was 127.00 mm × 6.35 mm × 12.70 mm. In the test, a load of 0.980 N was applied to the trabecular specimen, and the deflection of the trabeculars was measured by the displacement sensor in the system. After 240 s, the load was removed and the test stopped. Creep stiffness S at 60 s and slope creep rate m of the principal stiffness curve at 60 s were used to evaluate the low-temperature properties of the asphalt binder. Two sets of parallel samples were tested. The test instrument and asphalt samples are shown in Figure 3.

### 2.4. Calculation of SBS Content in Dry-Process Modifier Based on Infrared Spectrum

① Calculation Principle

The infrared spectroscopy method was applied for the determination of SBS content in an SBS modifier. The characteristic absorption peaks of the virgin asphalt and SBS modifier were first determined, the characteristic absorption peak area of the modified asphalt under different SBS modifier dosages was fitted, and then the SBS content of the sample to be measured was obtained according to the characteristic peak area ratio of the modified agent to be tested, which was the SBS content in the SBS modifier. In this experiment, characteristic absorption peaks under the 966 cm^−1^ and 1376 cm^−1^ wavenumbers were selected to represent the characteristic absorption peaks of SBS and virgin asphalt, respectively.

② Specimens

Standard samples: 3%, 4%, 5%, 6%, and 7% SBS-modified asphalt.

Samples to be tested: 5% and 7% Liaohe SBS-modified asphalt and 5% and 7% Guolu SBS-modified asphalt.

③ Curve Fitting and Content Calculation

**Figure 4:**
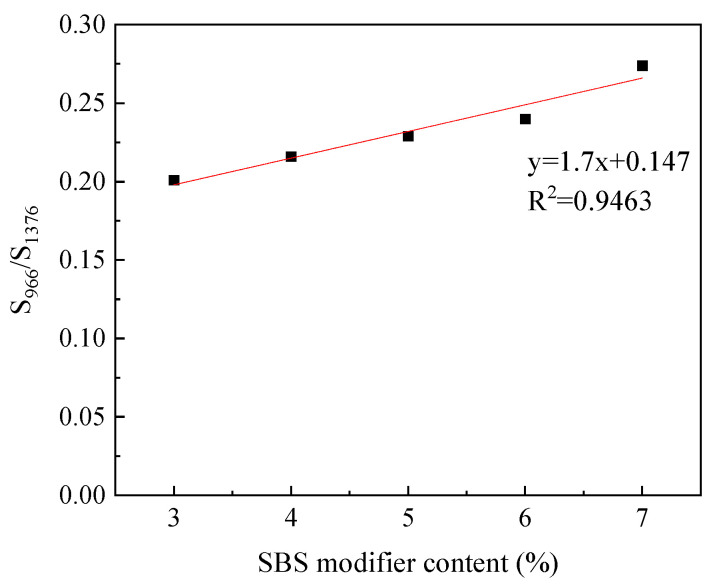
Standard curve of SBS content.

An amount of 90# virgin asphalt of Liaohe brand was used to prepare SBS-modified asphalt with a content of 3%, 4%, 5%, 6%, and 7%, respectively, and an infrared spectrogram at 4000–400 cm^−1^ wavenumber was retrieved; three sets of parallel samples were tested. Peak areas of 966 cm^−1^ and 1376 cm^−1^ were obtained, and then the peak area ratio S_966_/S_1376_ was calculated. The results are shown in Table 4, and the fitting curves are shown in Figure 4. 

The peak area ratio of the sample was substituted into a standard curve to obtain the SBS content in the sample to be measured, and the ratio of its content to the content of the dry-process modifier was the SBS content of the dry-process modifier. The final calculation results are as follows: the SBS content in the Liaohe brand dry-process modifier was 73.90%, and the SBS content in the Guolu brand dry-process modifier was 70.39%.

## 3. Basic Properties of Dry-Process SBS-Modified Asphalt Binder under Different Preparation Processes

### 3.1. Preparation Process of Dry-Process SBS-Modified Asphalt Binder

To study whether a dry-process SBS modifier can fully play the role of modification, and to study the performance index of a modified asphalt binder in a dry-process SBS-modified asphalt mixture to the greatest extent, it is necessary to obtain modified asphalt binder from a dry-process SBS-modified asphalt mixture by unconventional means. In the process of preparing a dry-process SBS-modified asphalt mixture, the modifier is melted and dispersed in virgin asphalt binder during mixing, and the aggregate plays the role of shearing and grinding. Because dry-process SBS modifier has the characteristic of easy melting if the modified asphalt binder is prepared by the traditional wet process, the melting effect and dispersion state of the dry-process SBS modifier cannot be reflected, so its action characteristics should be considered when preparing the modified asphalt binder, and the modifier should directly interact with the asphalt binder under the grinding action of the stone to obtain dry-process SBS-modified asphalt binder. Referring to relevant research [25] and the continuous adjustment of temperature and time used in actual tests, the preparation processes of two dry SBS-modified asphalt binders were preliminarily determined. The gradation and nature of the coarse aggregate used are shown in Table 5. The preparation and extraction process of dry SBS-modified asphalt binder is shown in Figure 2 and Figure 3.

**Figure 5:**
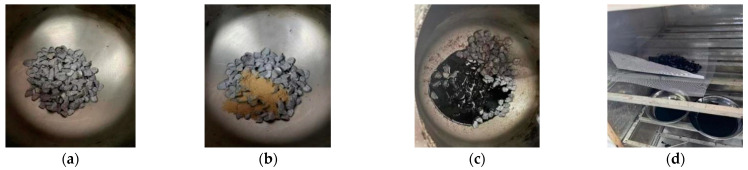
Plan 1. (**a**) Coarse aggregate. (**b**) Add modifier and stir. (**c**) Add virgin asphalt binder and stir. (**d**) Collect modified asphalt binder.

Plan 1: Preheat coarse aggregate with a particle size greater than 9.5 mm to 185 °C, pour it into a mixing pot at 180 °C, add dry-process SBS modifier to stir for 30 s, and then pour virgin asphalt binder into the mixing pot for 90 s. The mixed mixture is placed in an iron net and placed in an oven at 180 °C for 2 h to collect the asphalt binder dripping from the iron net. The detailed procedure is shown in Figure 5. 

**Figure 6:**
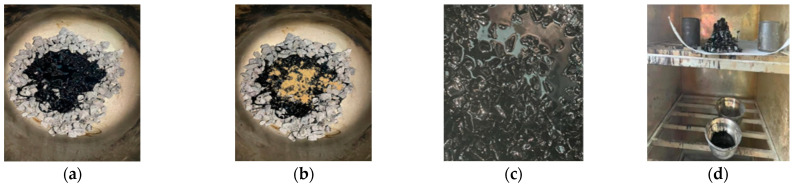
Plan 2. (**a**) Asphalt binder and aggregate. (**b**) Add modifier. (**c**) Mix 120 s. (**d**) Collect asphalt binder.

Plan 2: To obtain a dry-process SBS-modified asphalt binder that is closer to the real dosage, it is necessary to make full contact between the asphalt binder and the modifier. The preparation process of this plan is as follows: coarse aggregate with a particle size greater than 9.5 mm is preheated to 165 °C and poured into a mixing pot at 160 °C, and virgin asphalt binder and dry-process SBS modifier are poured into the mixing pot at the same time for stirring. To ensure uniform mixing, the mixing time is set to 120 s, and then the dry-process SBS-modified asphalt mixture after mixing is contained in an iron mesh and placed in an oven at 180 °C for 2 h; the asphalt binder dripping from the iron mesh is collected. The preparation process not only reduces the extrusion and friction effect of stone on the modifier during the preparation of the mixture but also enables the asphalt binder to be evenly mixed with the modifier, in order to obtain dry-process SBS-modified asphalt binder with close to the real content. The detailed procedure is shown in Figure 6. 

Through comparison, it is found that the dry-process SBS-modified asphalt binder prepared by plan 2 is more uniform, and a dry-process SBS-modified asphalt binder can be obtained with close to the real content. Referring to the “Test Regulations for Highway Engineering Asphalt and Asphalt Mixture” (JTG E20-2011), the basic indexes of the 5% dry-process SBS-modified asphalt binder prepared by the two plans were compared, and the results are shown in Table 6. In this paper, the modified asphalt binders prepared by Liaohe and Guolu brand dry modifiers are named ZT-L and ZT-G, respectively.

Table 6 shows that the basic indicators of plan 2 are significantly better than those of plan 1. Due to the large amount of oil and stone used in the preparation process and the large amount of modifier, a small amount of modifier clumping will occur during the mixing of modifier and stone in plan 1, resulting in insufficient modifier content in the prepared modified asphalt binder. In plan 2, because the asphalt binder is added before the modifier, the modifier will be melted and dispersed in the asphalt binder under the simultaneous action of asphalt binder and stone during the mixing process. Asphalt binder plays a role in inhibiting the formation of modifier agglomeration, and the modifier can fully contact with the asphalt so that the modifier content of the modified asphalt binder prepared by plan 2 is closer to the real dosage. 

Therefore, in this study, plan 2 was used to prepare dry-process SBS-modified asphalt binder, and then performance and microscopic tests of the asphalt binder were carried out.

### 3.2. Rheological Properties of Dry-Process SBS-Modified Asphalt with Different Dosages

According to the Determination of Rheological Properties of Asphalt (DSR Method), a DSR was used to measure the complex shear modulus G* and phase angle δ of the modified asphalt at a given temperature and load frequency. In this way, the viscoelastic properties of the modified asphalt were characterized. Firstly, the linear viscoelastic interval of each modified asphalt sample was determined by stress–strain scanning, and a frequency sweep, temperature sweep, multiple-stress creep recovery test, and linear amplitude sweep test were carried out on each modified asphalt sample in the linear viscoelastic interval. Two sets of parallel samples were tested in all experiments. 

#### 3.2.1. Frequency Sweep Test

**Figure 7:**
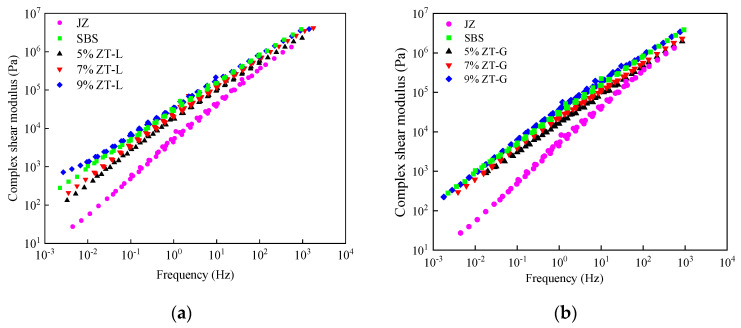
Main curves of complex modulus of different asphalt samples. (**a**) Different dosages of ZT-L. (**b**) Different dosages of ZT-G.

In order to characterize the viscoelastic properties of asphalt pavement under a dynamic load during service, a sweep test with a DSR was carried out to obtain the complex shear modulus G* and phase angle δ by continuously changing the loading frequency of the asphalt specimens. The complex shear modulus G* is the ratio of the maximum shear stress and shear strain of linear viscoelastic materials under a continuous sinusoidal load and is an evaluation index of total resistance. It consists of two parts: real and imaginary components, as shown in formula 1. Of these, G′ is the dynamic modulus of elasticity, that is, the elastic part, which reflects the energy stored during the deformation of asphalt. G″ is the loss modulus of elasticity, that is, the viscous part, which is equivalent to the loss modulus of elasticity produced by dynamic viscosity, reflecting the energy lost in the form of heat due to internal friction in the process of deformation of asphalt. Therefore, the measured complex shear modulus was constructed as a time–temperature-equivalent master curve with 48 °C as the reference temperature. The master curve of the modulus at any other temperature and frequency can be obtained by translating the displacement factor, and the results are shown in Figure 7.

G* = G′ + iG″(1)

where G*—complex shear modulus, G′—dynamic elastic modulus, and G″—loss of elastic modulus. 

It can be seen from Figure 7a,b that compared with virgin asphalt, the complex modulus of several modified asphalts has been significantly improved, and with an increase in the dosage, the complex moduli of two dry-process modified asphalts increase.

As can be seen from Figure 7a, the magnitude of the complex modulus is 9% ZT-L > SBS > 7% ZT-L > 5% ZT-L > JZ at low frequency. In the principle of time–temperature equivalence, low frequency represents high temperature, and the larger the complex modulus at a low frequency, the stronger the shear deformation resistance at a high temperature. At a high frequency, the performance of 9% ZT-L-modified asphalt is comparable to that of wet SBS-modified asphalt, and the complex modulus of the rest of the asphalt samples is the same as that at a low frequency. Overall, the shear deformation resistance of 9% ZT-L for asphalt was the most improved, and the wet SBS-modified asphalt was between 9% ZT-L-modified asphalt and 7% ZT-L-modified asphalt. 

Through Figure 7b, it can be found that the magnitude relationship of the complex modulus is 9% ZT-G > SBS > 7% ZT-G > 5% ZT-G > JZ, and the complex modulus of 9% ZT-G- and SBS-modified asphalt is comparable to that in the high-frequency and low-frequency stages. 

#### 3.2.2. Temperature Scan Test

**Figure 8:**
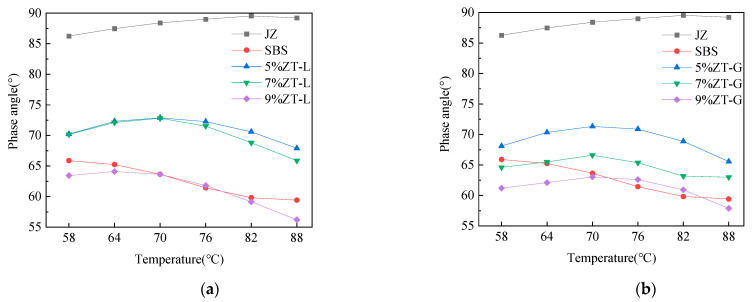
Phase angles of different asphalt samples. (**a**) Different dosages of ZT-L. (**b**) Different dosages of ZT-G.

To study the relationship between the viscoelastic properties of each asphalt and asphalt type, temperature, and modifier content, the viscoelastic state of each asphalt sample at different temperatures was analyzed through the phase angle obtained by temperature scanning. In this study, a temperature range of 58–88 °C was selected for scanning; the temperature interval was 6 °C, and the scanning frequency was 1.59 Hz. The phase angle and rutting factor of ZT-L-modified asphalt, ZT-G-modified asphalt, virgin asphalt, and wet SBS-modified asphalt were compared, respectively, and the results are shown in Figure 8.

By observing the phase angle of each asphalt sample, it can be found that the phase angle of virgin asphalt is the largest, and its phase angle gradually increases with an increase in temperature; the higher the temperature, the smaller the increase in the phase angle. When the temperature reaches 82 °C, the phase angle is close to 90 °C, indicating that the virgin asphalt is close to a viscous flow state at this temperature and almost loses the characteristics of elastomers. However, the performance of modified asphalt is very different from that of virgin asphalt, and it has a certain relationship with the content of modifier. The phase angle of SBS-modified asphalt decreases with an increase in temperature, while the phase angle of ZT-L and ZT-G under each content shows a trend of increasing first and then decreasing with an increase in temperature. 

Analyzing the reasons for the above phenomenon, a decrease in the phase angle of SBS-modified asphalt in the temperature-increase stage may be due to the contraction and curling of the SBS molecular chain under the condition of low temperature [26]; with an increase in temperature, the molecular chain gradually stretches, and the stretched SBS forms a three-dimensional network structure with the virgin asphalt, which offsets the effect of the increase in the viscous component of the virgin asphalt and makes the elasticity of the SBS-modified asphalt gradually increase. The reason why the phase angle of the two dry-process SBS-modified asphalts first increased and then decreased was that when the temperature was not high, the viscoelastic properties of the virgin asphalt dominated with the increase in temperature, and the increase in viscous components led to a gradual increase in the phase angle. However, when the temperature is high, the increase in the viscous component of the virgin asphalt will gradually decrease, and SBS will exert its high elasticity to gradually reduce the phase angle. 

**Figure 9:**
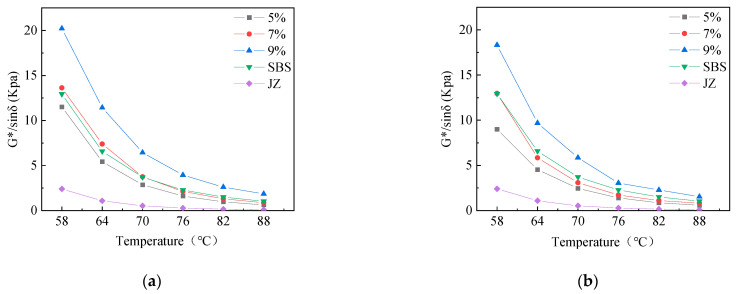
Rutting factors of different asphalt samples. (**a**) Different dosages of ZT-L; (**b**) different dosages of ZT-G.

To compare and analyze the high-temperature stability and rutting resistance of dry-process SBS-modified asphalt, the rutting factors of several asphalt samples were studied. The rutting factors of several asphalt specimens are shown in Figure 9. 

Compared with the rutting factor of SBS-modified asphalt, the rutting factor of ZT-L and ZT-G with 5% content was smaller. The rutting factor of the two dry-process SBS-modified asphalt was significantly larger than that of the wet SBS-modified asphalt at a 9% dosage, and the rutting factor of 9% ZT-L was the largest. For the two kinds of dry-process SBS-modified asphalt, the rutting resistance was the weakest when the content was 5%; the rutting resistance was comparable to that of the wet SBS-modified asphalt at a content of 7%; and the rutting factor was greater than that of the wet SBS-modified asphalt when the content was 9%, indicating that ZT-L and ZT-G with 9% content had the strongest rutting resistance. 

#### 3.2.3. Multiple-Stress Creep Recovery Test

**Figure 10:**
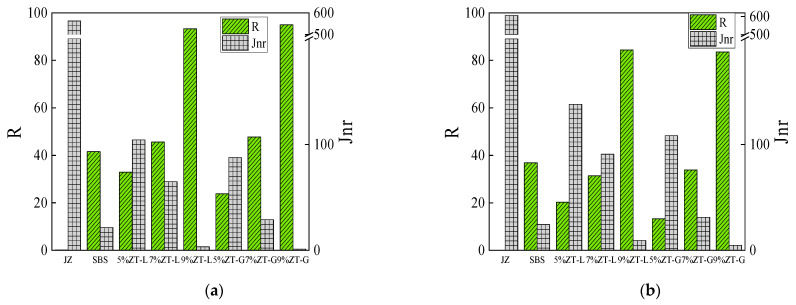
Elastic recovery rate and non-recoverable creep compliance of asphalt. (**a**) 0.1 kPa. (**b**) 3.2 kPa.

Multiple-stress creep recovery (MSCR) tests were used to analyze the high-temperature performance of dry-process SBS-modified asphalt. The stress levels of the test were 0.1 kPa and 3.2 kPa, and each stress condition consisted of 10 loading cycles, each loading cycle with constant stress for 1 s and unloading recovery for 9 s. According to the characteristics of asphalt, 60 °C was selected as the test temperature in the study, and the creep recovery test results of virgin asphalt and wet SBS-modified asphalt were compared with dry-process SBS-modified asphalt with different dosages; the test results are shown in Figure 10. 

Creep recovery rate (R) is used to characterize the ratio between the rebound deformation and the total deformation of asphalt materials at different stress levels to obtain the elastic properties of asphalt, and the larger the creep recovery rate, the better the high-temperature performance of asphalt. The non-recoverable creep compliance (Jnr) is used to represent the viscous residual deformation of asphalt materials, and the higher its value, the worse the high-temperature resistance of the material.

As can be seen from Figure 10, due to the poor high-temperature resistance of the virgin asphalt itself, the virgin asphalt has internal damage under a stress of 3.2 kPa, and the creep recovery rate of the virgin asphalt at 0.1 kPa is only 0.1%. Compared with virgin asphalt, the creep recovery rate of wet- and dry-process SBS-modified asphalt was significantly higher than that of virgin asphalt, and the non-recoverable creep flexibility was significantly reduced, indicating that the addition of SBS enhanced the rutting resistance of asphalt. In addition, compared with the non-recoverable creep compliance at 0.1 kPa and 3.2 kPa, the non-recoverable creep compliance at 3.2 kPa is larger, indicating that the asphalt pavement is more prone to rutting under heavy traffic loads. For ZT-L and ZT-G dry-process modified asphalt, with an increase in modifier content, the creep recovery rate of both of them increased significantly, and the non-recoverable creep compliance decreased significantly, indicating that the increase in modifier content enhanced the elasticity of the asphalt. Compared with wet SBS-modified asphalt, ZT-L and ZT-G with a content of 9% have a larger elastic recovery rate and a smaller non-recoverable creep flexibility. 

The MSCR results showed that the high-temperature performance of the two dry-process SBS-modified asphalts was significantly higher than that of the virgin asphalt, and the high-temperature performance improvement effect was more significant with the increase in modifier content. Compared with the wet SBS-modified asphalt, the creep recovery rate of the 7% dry-process SBS-modified asphalt is similar, but the non-recoverable creep flexibility is larger, and the stress sensitivity of the dry-process modified asphalt is poor, so the wet SBS-modified asphalt has better high-temperature performance. A comprehensive comparison of the high-temperature performance of ZT-L, ZT-G, and wet SBS-modified asphalt showed that ZT-L and ZT-G with 9% content had the best high-temperature performance; this was followed by wet SBS-modified asphalt, ZT-L, and ZT-G with 7% content, which was slightly worse than that of wet SBS-modified asphalt, and ZT-L and ZT-G with 5% content had the worst high-temperature performance.

#### 3.2.4. Linear Amplitude Sweep Test

A linear amplitude scanning test (LAS) was used to carry out a medium-temperature fatigue test on different bitumen samples. The LAS experiment is mainly divided into two stages. First of all, a frequency scanning test is carried out on the sample in the range of 0.1–30 Hz at a 0.1% strain level. Then an oscillating shear mode under a strain control mode is used at the selected temperature conditions with a loading frequency of 10 Hz, and the strain increases linearly from 0.1 to 30% during 3100 loads, recording the peak shear strain and peak shear stress. Then, the damage characteristic curve of the asphalt sample was obtained by the viscoelastic continuum damage model (VECD model). The vertical coordinate C represents the integrity parameter of asphalt, and the horizontal coordinate D represents the cumulative damage parameter. When C = 1, the asphalt sample is in an undamaged state. When C = 0, the asphalt sample has been completely destroyed. When the cumulative damage parameter D is given, the greater the C, the stronger the material’s resistance to fatigue damage.

**Figure 11:**
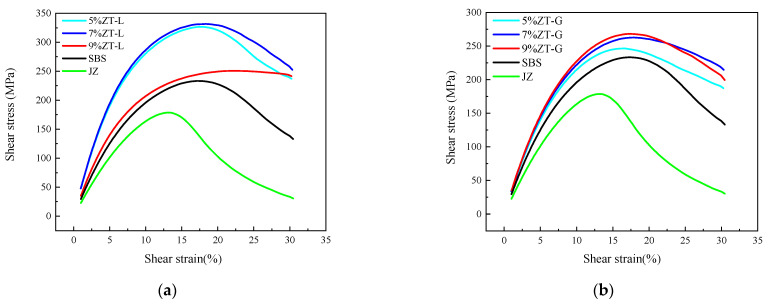
Stress–strain curve of different asphalt samples. (**a**) Different dosages of ZT-L. (**b**) Different dosages of ZT-G.

**Figure 12:**
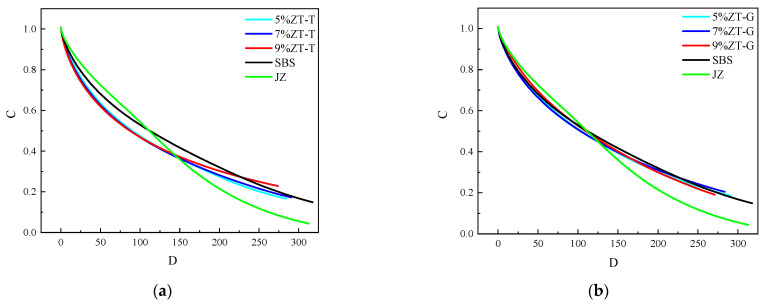
Fatigue damage curves of different asphalt samples. (**a**) Different dosages of ZT-L. (**b**) Different dosages of ZT-G.

Stress–strain curves of virgin asphalt, SBS-modified asphalt, and dry-process SBS-modified asphalt are plotted as shown in Figure 11, and a fatigue damage curve is shown in Figure 12.

The stress–strain curves of each asphalt sample are compared and analyzed in Figure 8. As can be seen from Figure 11a, the strain level of several modified asphalts at the time of initial failure is significantly greater than that of virgin asphalt, indicating that the addition of three modifiers reduces the dependence of asphalt on strain, and the width of the peak zone of 9%ZT-L is the largest, which proves that its strain sensitivity is the least. At the 5% dosage, there was no significant difference between the strain and peak area width of the two dry-process modified asphalt and the wet SBS-modified asphalt at the initial failure. As can be seen from Figure 11b, 5% ZT-G has the lowest dependence on strain, while 7% ZT-G and 9% ZT-G have no significant difference in strain during fatigue failure, and there is no significant difference in the width of the peak region. The shear stress of the two types of dry-process-modified asphalt is greater than that of the wet SBS-modified asphalt during fatigue failure. 

The fatigue damage curve of asphalt was analyzed. The slope of the fatigue damage curve is representative of the speed of damage. As can be seen from Figure 12, virgin asphalt has the largest slope and the largest damage velocity. Compared with wet SBS-modified asphalt, ZT-L has a faster damage rate in the early stage, but the damage rate slows down in the later stage, showing a different law from that of wet SBS-modified asphalt. The damage curve trend of ZT-G and wet SBS-modified asphalt is the same. The fatigue life calculation and estimation results of the LAS test are shown in Table 7.

Through Table 7, we find that the fatigue life of the three modifiers for virgin asphalt is greatly improved, and the different types of dry-process modifiers exhibit different rules. The 2.5% strain level characterizes a low-strength pavement, while the 5% strain characterizes a higher-strength pavement. For the fatigue life of each asphalt sample at a 5% strain level, the fatigue life of all modified asphalt samples is greater than that of virgin asphalt, and all of them are more than twice that of virgin asphalt. The fatigue life of ZT-L and ZT-G at a 5% dosage is slightly smaller than that of wet SBS-modified asphalt, the fatigue life of ZT-L and ZT-G at 7% dosage is comparable to that of wet SBS-modified asphalt, and the fatigue life of ZT-L and ZT-G at 9% dosage is significantly greater than that of wet SBS-modified asphalt. Of these, the fatigue life of 9% ZT-L is the largest, which is more than twice that of wet SBS-modified asphalt.

### 3.3. Rheological Test of Low-Temperature Bending Beam

(1) Stiffness modulus and creep rate

The S value is used to characterize the ability of asphalt materials to resist deformation at low temperatures under a constant leaching load, and the larger the S value, the more likely the asphalt is to crack. The higher the m value, the better the stress relaxation capacity and the better the crack resistance of the asphalt. The rheological test results of low-temperature bending beams of different asphalts are shown in Table 8. 

It can be found from Table 8 that the stiffness moduli of all modified asphalts at −12 °C, −18 °C, and −24 °C are lower than that of virgin asphalt, and the creep rates are greater than that of virgin asphalt. Virgin asphalt, although only at −12 °C, meets the specification requirements of S < 300 MPa and m > 0.3, while ZT-L, ZT-G, and wet SBS-modified asphalt meet the specification requirements at −12 °C and −18 °C, but do not meet the specification requirements at −24 °C. 

The addition of SBS decreases the stiffness modulus of asphalt and increases the creep rate. The decrease in stiffness modulus indicates that the low-temperature flexibility of asphalt is improved, and the creep rate increases indicate that the crack resistance and stress relaxation ability of asphalt are enhanced. With an increase in the modifier content in ZT-L and ZT-G, the S value decreases gradually, while the m value increases gradually. At −18 °C, a low-temperature creep performance comparable to that of wet SBS-modified asphalt can be achieved at a dosage of 7% for the two dry-process SBS-modified asphalts. The results show that the addition of SBS gives the asphalt better low-temperature deformation ability and makes it not prone to brittle fracture failure, and dry-process SBS can effectively enhance the low-temperature creep performance of asphalt.

(2) Research on low-temperature performance based on Burgers model

Based on analyzing the stiffness modulus and creep rate, Burgers model was used to fit the creep compliance of asphalt to study the viscoelastic constitutive relationship of asphalt specimens. The creep compliance of each asphalt sample at −18 °C was fitted, and the relaxation time *λ*=$\eta_{1}$/*E*_1_ was calculated; the results are shown in Table 9.

**Figure 13:**
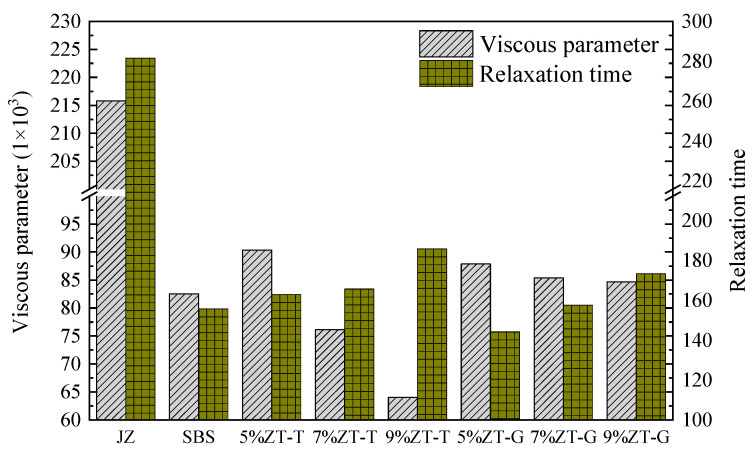
Viscosity coefficient and relaxation time of each sample.

As can be seen from Table 9, when fitting BBR test results with the Burgers model, the correlation coefficients are all above 0.99. In terms of values, $\eta_{1}$ and $\eta_{2}$ are at least 100 times larger than E_1_ and E_2_, indicating that the viscosity characteristics of asphalt material in the Burgers model are much greater than the elasticity characteristics. The viscosity coefficient and relaxation time of each sample are shown in Figure 13.

Among the four parameters of the Burgers model theory, the viscosity coefficient η_1_ determines the deformation ability of asphalt, and the larger the η_1_, the stronger the deformation ability of the asphalt. The η_1_ of virgin asphalt is significantly greater than that of other modified asphalt, and the corresponding low-temperature deformation ability is inferior to that of other modified asphalt. With an increase in dosage, the η_1_ of ZT-L gradually decreased, indicating that the low-temperature crack resistance of ZT-L gradually increased in the range of 5% to 9%. The low-temperature crack resistance of ZT-L with 7% and 9% content is better than that of wet SBS-modified asphalt. However, ZT-G is different, and the η_1_ under the three dosages is not much different: it is slightly larger than that of wet SBS-modified asphalt.

Relaxation time λ represents the dissipation ability of asphalt to a load, which can reflect the trend of the internal stress of the asphalt with time. The smaller the λ, the faster the stress dissipates and the less likely the asphalt is to crack. It can be found that the λ of virgin asphalt is the largest, and the relaxation time of ZT-L and ZT-G gradually increases with an increase in modifier content. The main reason is that with an increase in the amount of dry-process SBS modifier, the fluidity of the asphalt gradually deteriorates and stress concentration occurs, which leads to the slowing down of stress dissipation.

Based on the experimental results, it was found that the higher the content of ZT-L, the more significant the improvement of low-temperature performance, while the relationship between the content and performance of ZT-G was weaker. On the whole, the improvement of the low-temperature performance of the ZT-G modifier is the worst of the three modifiers, while the improvement of the low-temperature performance of ZT-L is more obvious, and the improvement of wet SBS-modified asphalt is in between. 

To summarize the above test results, the test results of the rutting factor and MSCR in the high-temperature evaluation system show that the high-temperature performance of ZT-L and ZT-G at 7% content is slightly worse than that of wet SBS-modified asphalt. The results of the linear amplitude scanning (LAS) test showed that the fatigue life of the three was similar; the fatigue life of ZT-G was slightly higher, followed by the fatigue life of wet SBS-modified asphalt, and the fatigue life of ZT-L was slightly lower, but there was little difference in the fatigue life of the three. The BBR test results show that the low-temperature creep performance of ZT-L has a strong relationship with the dosage, and 7% ZT-L exhibits better low-temperature crack resistance than wet SBS-modified asphalt, while 7% ZT-G has a slightly worse low-temperature performance than wet SBS-modified asphalt. ZT-L and ZT-G with 5% content had the best high- and low-temperature and fatigue properties.

## 4. Research on the Modification Mechanism of Dry-Process SBS-Modified Asphalt

### 4.1. Characteristic Functional Group Analysis

**Figure 14:**
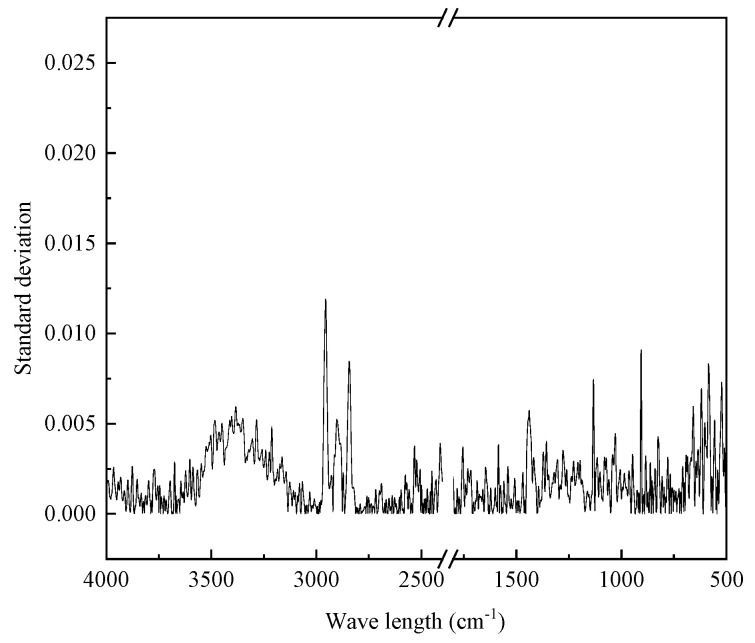
Standard deviation of parallel samples.

**Figure 15:**
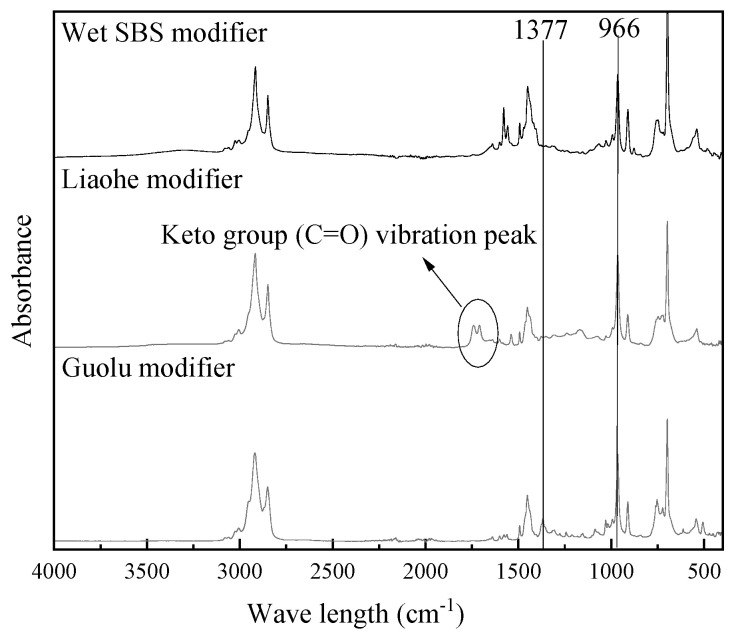
Infrared spectra of the three modifiers.

Infrared spectroscopy tests were carried out on wet SBS modifier, ZT-L modifier, and ZT-G modifier at a room temperature of 23 °C, and two sets of parallel tests were set for each sample. Taking conventional SBS modifiers as an example, the standard deviation between parallel samples is shown in Figure 14. The test results of different modifiers are shown in Figure 15.

It can be seen from Figure 14 that the infrared spectral curve is within 0.01 standard deviation, and the two curves have a high coincidence, so the average value of the measured results has a high accuracy, and the absorbance of a certain wave-number position can reflect the content of functional groups represented by this position in the material. Comparing the three modifiers, it can be found that all three have characteristic absorption peaks that can be used to characterize SBS, which is the unconjugated carbon-carbon double bond (C=C) vibration peak at 966 cm^−1^. The Liaohe modifier has a unique ketone group (C=O) vibrational peak at 1740 cm^−1^ and 1709 cm^−1^ wavelengths. The ketone group is a component of functional groups such as aldehydes, ketones, and carboxylic acid derivatives; ketone groups can undergo addition reactions and reduction reactions, and the oxygen atom of aldehydes and ketones can form hydrogen bonds with water and low-level aldehydes and can react with water. Ketones can inhibit oxidation reactions, which is helpful for improving the anti-aging performance of asphalt. It is speculated that the reason for the appearance of this functional group is that the Liaohe modifier contains substances that can enhance the water solubility of the modifier, which can make the modifier more effectively melt and disperse in the asphalt. The unique 1367 cm^−1^ methyl(-CH3-) umbrella vibration peak of the Guolu modifier is speculated to be related to the distillate component contained in the Guolu modifier. 

**Figure 16:**
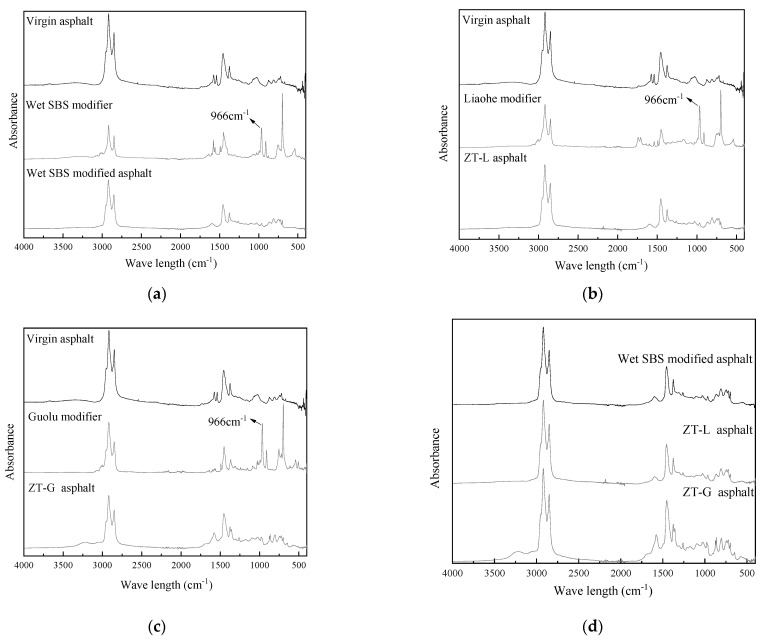
Infrared spectra of different samples. (**a**) Virgin asphalt, wet modifier, and wet modified asphalt. (**b**) Virgin asphalt, Liaohe modifier, and ZT-L asphalt. (**c**) Virgin asphalt, Guolu modifier, and ZT-G asphalt. (**d**) Different types of modified asphalt.

Infrared spectroscopy tests were carried out on different modifiers and modified asphalt, as shown in Figure 16a–d.

It is found that the main peak positions of wet SBS-, ZT-L-, and ZT-G-modified asphalt are the same. Characteristic peaks at 1377 cm^−1^ and 810 cm^−1^ of virgin asphalt and 966 cm^−1^ and 699 cm^−1^ of SBS were obvious.

After the addition of wet SBS and Guolu modifiers to virgin asphalt, the characteristic peaks of the virgin asphalt, the modifier, and the modified asphalt are basically in a simple superimposed state, which proves that the two modifiers and the asphalt mainly have a physical blending effect. However, the Liaohe modifier has a ketone group (C=O) vibration peak, but the characteristic peak of ZT-L-modified asphalt disappears here because the ketone group reacts under acidic conditions to form acid or alcohol, which proves that there is a small amount of chemical reaction between Liaohe modifier and the virgin asphalt modification process. In addition, the presence of polar ketone groups in the Liaohe modifier makes it interact better with the aggregates with polar surfaces. This usually results in an increase in the viscosity/stiffness of the sample. This verifies the higher reliability of ZT-L asphalt than ZT-G asphalt modulus in Figure 7.

The absorption band of the sulfoxide group near 1033 cm^−1^ can characterize the degree of aging of asphalt. Compared with the virgin asphalt and the three modified asphalts, the modified asphalt has a lower absorption peak here, indicating that the SBS-modified asphalt prepared by wet and dry modification processes is almost unaffected by aging.

### 4.2. Study of the Distribution Characteristics of Dry-Process SBS Modifiers Based on Fluorescence Microscopy

**Figure 17:**
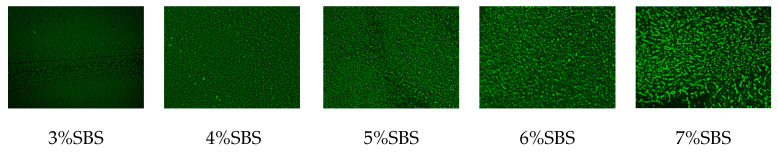
FM images of wet-modified asphalt with different modifier contents (Magnification = 400×).

**Figure 18:**
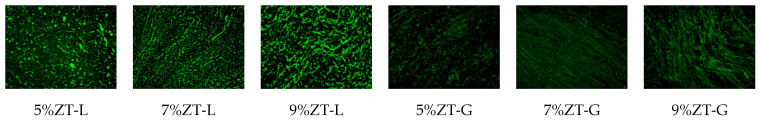
FM images of dry-process modified asphalt with different modifier contents (Magnification = 400×).

To study the melting and dispersion state of the modifier in asphalt under the dry-process modification process, the asphalt samples were observed by a fluorescence microscope. Python was used to perform grayscale processing, image threshold segmentation, morphological processing, and other steps of fluorescence imaging, and finally, the area of the modifier was mathematically counted by using the processed binary value map, and then the degree and uniformity of melt diffusion were analyzed. The observed test samples include 3–7% wet SBS-modified asphalt and 5%, 7%, and 9% Liaohe and Guolu dry-process SBS-modified asphalt, as shown in Figure 17 and Figure 18. 

Through the above fluorescence microscope images, it can be found that different modifier types, different preparation processes, and different modifier dosages have different degrees of influence on the distribution characteristics of modifiers in asphalt. Of these, wet SBS was dense and evenly distributed in spots, and the spots were of different sizes. When the dosage was less than 7%, the larger the dosage, the denser the distribution of spots, and the proportion of large particle spots increased. When the dosage reached 7%, SBS aggregated and distributed in a band-like manner. The distribution of SBS in ZT-L was a mixed distribution of dots and stripes, which had a strong relationship with the dosage. When the dosage was small, the shape of SBS was mostly speckled, and the size was different. When the dosage reached 9%, SBS aggregated and was distributed in a coarse network. The SBS in ZT-G was distributed in a fine network without spots, and the network structure became more and more dense with the increase in content. 

In general, the distribution of several modifiers in asphalt is very different. The main reason for this difference is that the dry-process SBS modifier directly interacts with asphalt in the form of melt decomposition, which has a short action time and less external intervention. The wet modification process is that after stirring, shearing, and other steps, the modifier and the asphalt fully swell, so that the modifier particles are smaller and the distribution in the asphalt is more uniform.

**Figure 19:**
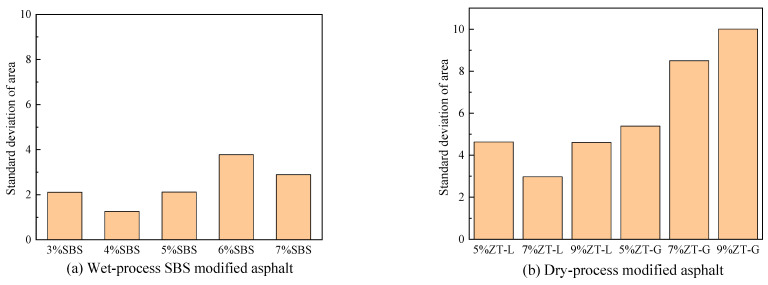
Area standard deviation of different modified asphalts.

The treated binary map was divided into 5 × 5 = 25 regions, the area proportion of modifiers in each region was calculated, and the uniformity of the dispersion of modifiers in each sample was analyzed by the variance of the area proportion. The results are shown in Figure 19. 

Through Figure 19, we can see that the uniformity of wet SBS-modified asphalt is the best, and the standard deviation range is between 1 and 4. The uniformity of the ZT-L is slightly poor, with standard deviations ranging from 2 to 5. The uniformity of ZT-G was the worst, with a standard deviation range of 5–10, and the standard deviation of the area proportion of ZT-G increased with an increase in the dosage, indicating that the uniformity of the distribution of the ZT-G modifier deteriorated with an increase in the dosage. Overall, for the two dry SBS-modified asphalts, ZT-L has better dispersion uniformity and better performance.

## 5. Conclusions

(1) In the process of preparing a dry-process SBS-modified asphalt mixture, the aggregate plays a role of shearing and grinding. Of the mixing method of adding the modifier first and then adding asphalt binder, and the mixing method of adding asphalt binder and modifier at the same time, the basic performance of the asphalt extracted from the latter is better.
(2) The performance of the two dry-process SBS-modified asphalts is enhanced with an increase in the dosage. The high- and low-temperature properties and fatigue properties of the two dry-process SBS-modified asphalts at a 7% dosage are close to those of the wet SBS-modified asphalt at a 5% dosage.
(3) The mechanism of action of the two modifiers is mainly the physical blending of SBS and asphalt binder, but due to the incorporation of oil, crosslinker, solubilizer, and other substances into the dry-process SBS modifier, there is a small amount of chemical reaction with asphalt binder in the melting process.
(4) Under the wet and dry modification processes, the distribution states of SBS modifiers in asphalt are different. The distribution state of SBS in asphalt is slightly worse under the dry modification process, but the dry modification process can accurately supervise the quality of SBS-modified asphalt and its mixture, reduce the cost of production equipment and transportation, and solve the problems of conventional wet modified asphalt segregation. It has certain application prospects and research value. For the two kinds of dry SBS-modified asphalt, ZT-L has better dispersion uniformity and better performance. In practical engineering applications, the comprehensive application efficiency of modified asphalt binder under different modification processes and different modifiers should be considered.

## Figures and Tables

**Table 1:** Technical index of Liaohe 90# virgin asphalt binder.

Asphalt Binder	Test Index	Value	Testing Regulations
Liaohe 90#	Penetration (25 °C, 100 g, 5 s) (0.1 mm)	80.2	T0604-2011 [20]
	Softening point (°C)	48.0	T0606-2011 [20]
	Ductility (15 °C, 5/min) (cm)	171	T0605-2011 [20]
	Density (g/cm^3^)	1.02	T0603-2011 [20]

**Table 2:** Basic technical specifications of wet-process SBS modifiers.

Type	Technical Index	Test Result	Testing Regulations
YH-791	Structure type	Linetype	--
	Block ratio S/B (%)	30/70	ASTM D-1416 [21]
	Tensile strength (≥MPa)	15.0	ASTM D-638 [22]
	Melt flow rate (g/10 min)	0.1–5.0	ASTM D-1238 [23]
	Elongation at break (≥%)	700	ASTM D-638 [22]

**Table 3:** Basic technical specifications of two dry-process SBS modifiers.

Technical Index	Unit	Liaohe	Guolu	Standard Requirement	Test Method
Appearance	-	Uniform particle	Uniform particle	Uniform particle	Visual inspection
SBS content	%	80	About 70%	≥50	T/CHTS20003-2018 Appendix B [24]
Ash content	%	1.3	1.2	≤5.0	JTG E20 T 0614 [25]
Dry mix dispersibility	-	No particle residue	No particle residue	No particle residue	Dry mix 1 min for visual inspection

**Table 4:** Peak area ratio of different SBS content.

Standard Samples	S_966_/S_1376_
3%SBS	0.201
4%SBS	0.216
5%SBS	0.229
6%SBS	0.24
7%SBS	0.274

**Table 5:** Aggregate gradation and nature for extracting SBS-modified asphalt binder by dry process.

Test Project	Unit	Coarse Aggregate Type		Canonical Value
		10–20 mm	5–10 mm	
Apparent density	g/cm^3^	2.742	2.726	≥2.6
Water absorption	%	0.54	0.42	≤2.0
Adhesiveness	-	level 5	level 5	no less than level 5

**Table 6:** Basic indexes of dry-process SBS-modified asphalt binder prepared by the two plans.

Technology	Asphalt Binder Sample	Penetration (0.1 × mm)	Softening Point (°C)	Ductility (cm)
Testing regulations	/	T0604-2011	T0606-2011	T0605-2011
Plan 1	5%ZT-L	65.6	55.4	16.5
	5%ZT-G	69.8	51.6	18.9
Plan 2	5%ZT-L	59.5	61.7	31.2
	5%ZT-G	62.2	60.2	32.3

**Table 7:** Fatigue life calculation and estimation results of each asphalt sample.

Samples	Content	A	B	N_f 2.5_	N_f 5_
JZ	—	1.804 × 10^5^	2.498	18,290	3238
SBS	5%	9.214 × 10^5^	2.892	65,129	8776
	5%	6.669 × 10^5^	2.757	53,316	7886
ZT-L	7%	8.546 × 10^5^	2.704	61,945	8432
	9%	2.688 × 10^6^	3.352	157,294	18,279
	5%	7.063 × 10^5^	2.809	53,837	7681
ZT-G	7%	7.491 × 10^5^	2.757	69,890	8859
	9%	1.595 × 10^6^	3.307	95,434	11,339

Note: A—fatigue impedance, representing the ability of material to resist fatigue damage; B—load sensitivity; N_f_—fatigue life parameter; N_f 2.5_—the number of loading times when the initial modulus is reduced to 25%; N_f 5_—the number of loading times when the initial modulus is reduced to 50%.

**Table 8:** Stiffness modulus and creep rate of asphalt.

Samples	Content	Stiffness Modulus of 60 s/MPa			Creep Rate/m		
		−12 °C	−18 °C	−24 °C	−12 °C	−18 °C	−24 °C
JZ	-	126.6	441.3	642.1	0.335	0.243	0.174
SBS	5%	77.6	187.6	320.5	0.438	0.325	0.243
ZT-L	5%	84.8	210.7	513.9	0.411	0.315	0.222
	7%	78.6	175.7	342.0	0.412	0.328	0.234
	9%	71.9	133.5	316.0	0.424	0.346	0.241
ZT-G	5%	80.0	219.6	428.7	0.393	0.324	0.218
	7%	75.5	196.2	373.9	0.404	0.329	0.237
	9%	67.8	186.4	257.2	0.426	0.343	0.156

**Table 9:** Burgers model parameters of asphalt samples.

Samples	Content	E_1_	E_2_	$\eta_{1}$	$\eta_{2}$	λ	Correlation Coefficient R^2^
JZ	/	1021.59	1285.7	215,785.81	46,429.74	211.23	>0.99
SBS	5%	529.83	510.26	82,513.29	15,737.25	155.74	>0.99
	5%	554.59	593.37	90,358.55	20,598.16	162.93	>0.99
ZT-L	7%	459.22	394.06	76,124.46	16,300.12	165.77	>0.99
	9%	344.3	357.56	64,023.90	13,191.93	185.95	>0.99
	5%	609.27	613.95	87,870.85	19,954.81	144.22	>0.99
ZT-G	7%	541.7	527.7	85,370.79	20,749.98	157.60	>0.99
	9%	488.28	530	84,663.88	16,911.12	173.39	>0.99

Note: E_1_—elastic coefficient, E_2_—coefficient of delayed elastic, $\eta_{1}$—viscosity coefficient, $\eta_{2}$—coefficient of delayed viscosity.

## Data Availability

Data are contained within the article.
